# Proteolysis-Targeting Chimera (PROTAC): Is the Technology Looking at the Treatment of Brain Tumors?

**DOI:** 10.3389/fcell.2022.854352

**Published:** 2022-02-15

**Authors:** Ludovica Lospinoso Severini, Francesca Bufalieri, Paola Infante, Lucia Di Marcotullio

**Affiliations:** ^1^ Department of Molecular Medicine, University of Rome La Sapienza, Rome, Italy; ^2^ Istituto Pasteur-Fondazione Cenci Bolognetti, University of Rome La Sapienza, Rome, Italy

**Keywords:** protac (proteolysis targeting chimera), ubiquitylation (ubiquitination), cancer, glioblastoma, cancer therapy

## Abstract

Post-translational modifications, such as ubiquitylation, need to be tightly controlled to guarantee the accurate localization and activity of proteins. Ubiquitylation is a dynamic process primarily responsible for proteasome-mediated degradation of substrate proteins and crucial for both normal homeostasis and disease. Alterations in ubiquitylation lead to the upregulation of oncoproteins and/or downregulation of tumor suppressors, thus concurring in tumorigenesis. PROteolysis-TArgeting Chimera (PROTAC) is an innovative strategy that takes advantage by the cell’s own Ubiquitin-Proteasome System (UPS). Each PROTAC molecule is composed by a ligand that recruits the target protein of interest (POI), a ligand specific for an E3 ubiquitin ligase enzyme, and a linker that connects these units. Upon binding to the POI, the PROTAC recruits the E3 inducing ubiquitylation-dependent proteasome degradation of the POI. To date, PROTAC technology has entered in clinical trials for several human cancers. Here, we will discuss the advantages and limitations of PROTACs development and safety considerations for their clinical application. Furthermore, we will review the potential of PROTAC strategy as therapeutic option in brain tumor, focusing on glioblastoma.

## Introduction

### The Ubiquitin-Proteasome System

**FIGURE 1:**
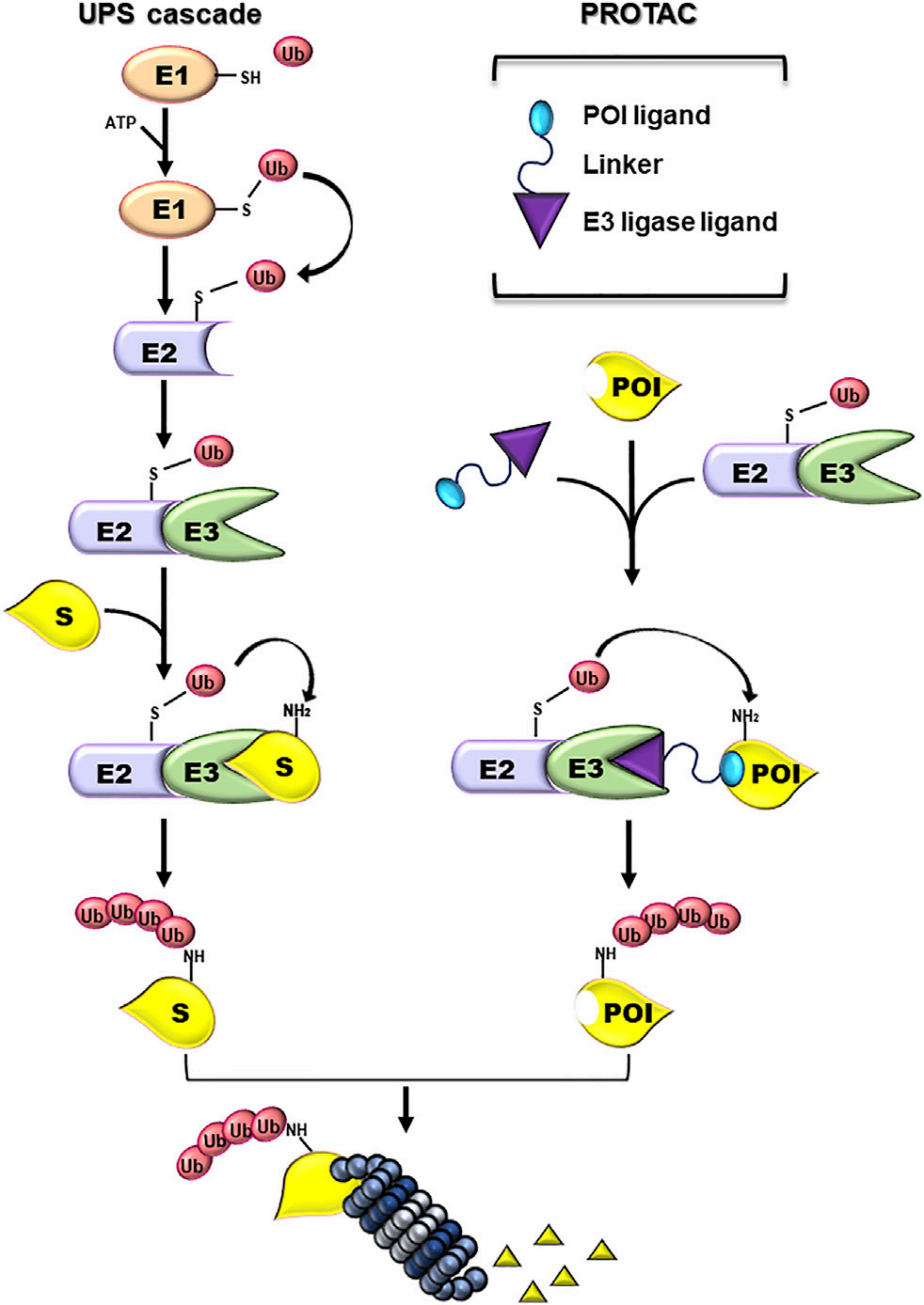
Ubiquitin-proteasome and PROTAC systems. Schematic representation of the enzymatic cascade of the Ubiquin-Proteasome System (UPS cascade; left side). Ubiquitylation is triggered by the ATP-dependent activation of the ubiquitin by E1 activating enzyme. Next, the ubiquitin (Ub) is bound to the E2 Ub-conjugating enzyme and, subsequently, transferred to a Lys residue on a substrate protein (S) by an E3-Ub ligase (E3). The formation of a poly-Ub chain, formed by more than four Ub moieties, can lead to the degradation of the substrate by the proteasome. PROTAC components and their mechanism of action (PROTAC; right side). PROTACs are heterobifunctional small molecules consisting of a ligand specific for the protein of interest (POI) and another ligand for E3, connected by a linker. PROTACs work by recruiting an E3 ligase into proximity of a specific POI that can be tagged with Ub and degraded by the proteasome.

The Ubiquitin-Proteasome System (UPS) is a cellular mechanism essential for maintaining the correct balance of protein turnover and cell homeostasis (Finley 2009; Hipp et al., 2019). UPS machinery includes chaperones and components of the proteolytic system (Kim et al., 2013): the first are required for an accurate protein folding; the latter converge on the 26S proteasome and guarantee the removal of unfolded and/or damaged proteins. To be targeted for proteasome-mediated degradation, proteins are covalently tagged with ubiquitin (Ub) moieties. This event requests the consequential activity of three enzymes: E1 Ub-activating enzyme (E1), E2 Ub-conjugating enzyme (E2), and E3 Ub-ligase (E3) (Kliza and Husnjak, 2020). First, an Ub molecule is activated by E1 in an ATP-dependent manner resulting in an E1-Ub conjugate. Then, a trans-thioesterification reaction allows the transfer of a molecule of Ub from E1 to E2. Lastly, an E3 binds at the same time the E2-Ub conjugate and the target protein favouring the transfer of Ub from the E2 to the substrate, directly or indirectly depending on the E3 family involved in the event (Infante et al., 2019; Sharma et al., 2021). Both the number of Ub moieties and the lysine linkage of Ub-Ub conjugation determine the fate of the protein (Welchman et al., 2005). Ub-tagged substrates are mostly addressed to the proteasome for degradation (Figure 1).

The UPS is finely regulated by E3 ligases that confer specificity of ubiquitylation through the recognition of substrates, thus making these enzymes considerable druggable targets. So far, several small molecule inhibitors (SMIs) have been designed to hit E3s. For instance, Mouse double minute two homolog (Mdm2), the E3 responsible of the ubiquitylation and degradation of p53**,** is highly expressed in sarcomas and breast cancers (∼20 and ∼15%, respectively) (Karni-Schmidt et al., 2016; Oliner et al., 2016) and represents a significant drug target in these tumors. Nutlin-3a, a small inhibitor of Mdm2, binds the hydrophobic pocket at the N-terminal of Mdm2 necessary for its binding with p53, preventing Mdm2-p53 interaction and activating p53 oncosuppressor functions in malignant cells (Vassilev et al., 2004).

SMIs present some inevitable limitations, including the possibility to target only a moderate percentage (∼20%) and an exiguous class, mainly enzymes, of human proteins (Schapira et al., 2019). Since most of disease-driven proteins are not enzymes, they are considered unconventional therapeutic targets. The urgent need to develop new strategies to target the undruggable proteome led to advances in antibody therapy (Jenkins et al., 2018; Dobosz and Dzieciątkowski 2019), although the difficulty to hit intracellular proteins still strongly limits the use of this option. The current emerging and successful strategy to target proteome is PROteolysis TArgeting Chimera (PROTAC) technology (Sakamoto et al., 2001; Schapira et al., 2019).

PROTACs take advantage of cell’s own UPS machinery to specifically address a protein of interest (POI) towards a proteasome-mediated degradation (Sakamoto et al., 2001).

### PROTAC Technology: The Two Side of the Coin

PROTACs are heterobifunctional molecules formed by two ligands connected by a linker. The first ligand (warhead) interacts with the POI, a different one binds with an E3, and the linker connects them (Figure 1) (An and Fu 2018). The proximity between the E3 and the POI mediated by PROTAC favors the ubiquitylation and catalyzes the degradation of the POI by the UPS.

PROTAC compounds have been developed more than 20 years ago (Sakamoto et al., 2001) and many efforts have been made in these 2 decades to improve their effectiveness. For example, peptide ligands in PROTAC structure have been modified in small molecules to ameliorate cell permeability (Schneekloth et al., 2008).

PROTACs show multiple advantages as compared to traditional SMIs, alongside several limitations. A PROTAC molecule can catalyse the degradation of multiple POI molecules, and its pharmacological effect is achieved at very low dosages compared to SMIs, thus reducing the toxicity. Of note, proteins considered as “undruggable” could be potentially targeted by PROTACs. This is relevant especially for transcription factors (TFs) involved in the progression of several malignancies (Bai et al., 2019; Zhou et al., 2019). For example, genomic alterations in c-MYC, FOXO1 or the androgen receptor (AR) have been described in neuroblastoma, breast, and prostate cancer, respectively (Bushweller 2019; Yu et al., 2019). Counteracting their expression through protein degradation represents a therapeutic strategy for these human malignancies. In this regard, two PROTACs targeting the AR and estrogenic receptor (ER) have reached the clinical practice in two phase I studies for the treatment of prostate and ER-positive breast cancer, respectively (Mullard 2019), sustaining the results obtained in this field.

Additionally, PROTACs can overcome SMIs resistance by targeting mutated POIs (Burslem et al., 2018; Zhao et al., 2019; Gonzalez et al., 2020), as well as the resistance resulting from POIs upregulation (Kregel et al., 2020).

However, some safety concerns associated with PROTACs need to be taken into consideration before supporting their entry in clinical practice. PROTACs limitations are mainly due to on-target and off-target toxicities. The on-target toxicities are related to the physiological functions of POI. Some proteins (i.e., kinases) hold enzymatic as well as scaffold functions, becoming essential for normal cellular functions. SMIs block only the enzymatic activity of POI, while the complete degradation induced by PROTACs interferes with both enzymatic and scaffolding function, eliciting undesirable consequences (Cromm et al., 2018; Nunes et al., 2019). Moreover, unlike SMI that can only partially inhibit the functions of their targets, a potent PROTAC can completely deplete its POIs. The partial inhibition consequent to SMIs treatment may be tolerable, while PROTAC-induced degradation could be harmful if POIs have essential functions for cell survival (Winter et al., 2015). The extent of cellular damage depends on the rate of the depleted protein resynthesis (Chan et al., 2018; Cromm et al., 2018; Olson et al., 2018; Testa et al., 2018; Smith et al., 2019). In addition, the inhibition of POIs mediated by SMIs is transient as opposed to the prolonged depletion PROTAC-mediated. In this case, the cellular/tissue context and the target features impact on the benefits or drawbacks of PROTACs. If a POI has redundant function in normal tissues, its prolonged degradation couldn’t be devastating for cells (Mason et al., 2007; Eichhorn et al., 2014; Khan et al., 2019). On the contrary, targeting a POI indispensable for physiological cellular activities can cause on-target toxicities.

Off-target toxicities often arise from the “unintentional” degradation of proteins. This event may occur when the non-target protein is not directly bound to the PROTAC but is in complex with the POI or in its proximity (Hsu et al., 2020). Since PROTACs form a ternary complex between POI and E3, a phenomenon known as “Hook effect” can take place. In particular, the formation of the ternary complexes is inhibited with high PROTACs concentrations causing an excess of binary bindings PROTAC-POI or PROTAC-E3, thus invalidating target degradation (Pettersson and Crews 2019). Furthermore, the generation of PROTAC-E3 binary complexes can induce the degradation of lower-affinity non-targeted proteins (Moreau et al., 2020). This event may affect substrates essential for cellular homeostasis (Schmitt et al., 2002), or may cause the accumulation of off-target ubiquitylated proteins saturating the UPS and dysregulating the proteostasis.

### PROTACs Optimization Strategies

PROTAC is a relatively new research field with rapid developments that, however, still needs laborious optimization. Biological and physical-chemical properties of this technology can be fine-tuned. The linker length is a crucial structural element that can be improved. Too short linkers may cause a steric clash that disrupts ternary complex, thus impairing PROTAC activity. Conversely, too long linkers can give two heads of a PROTAC more motility, thus changing molecule stability. Moreover, an excessive linker length increases the molecular weight and reduces cell permeability of a PROTAC.

The first linker used in PROTAC design has been a flexible one, such as polyethylene glycol (PEG), which improves water solubility (Bai et al., 2019; Khan et al., 2019) or polymethylene chains. Recently, “click chemistry” based on coppercatalyzed azide-alkyne cycloaddition (CuAAC) and the Diels–Alder (DA) reaction has been applied in PROTAC preparation (Wang et al., 2020). The resulting PROTACs can be faster validated for their degradation capability and can self-assembly as active molecules in live cells (Lebraud et al., 2016).

The rigidity of the linker represents another important aspect that impacts on pharmacokinetic properties and oral bioavailability of PROTACs (Farnaby et al., 2019; Testa et al., 2020). Nevertheless, the design of an optimal rigid linker could be difficult if the cocrystal structure of the ternary complex is unknown.

The human genome encodes for more than 600 E3s, but only 1% of them have been explored for substrate degradation (Khan et al., 2020). Since E3s define target specificity, this feature could be useful to increase efficacy and decrease toxicity of the PROTACs. For example, one PROTAC optimization strategy is based on E3 specific expression in tissues (i.e. the F-box and leucine-rich repeat protein 16, FBXL16, is specifically expressed in cerebral cortex (Clifford et al., 1999)) and/or cellular compartments (i.e. the DDB1- and CUL4-associated factor 16, DCAF16, localizes only in the nucleus (Robb et al., 2017).

### PROTACs in Human Cancers

Cancer is a multistep process characterized by abnormal cellular proliferation and dissemination due to genomic and epigenomic alterations (Hanahan and Weinberg 2011). The identification of molecular alterations involved in the oncogenic features has become attractive for the development of novel therapeutics (Ocaña et al., 2018). The clinical use of proteasome inhibitors in oncology demonstrates how the disbalance in protein homeostasis reflects an oncogenic vulnerability in some malignancies (Inobe and Matouschek 2014; Schapira et al., 2019). Indeed, an accurate proteostasis is crucial in cells characterized by a high rate of protein turnover, such as tumor cells, that consequently need a very efficient and quick protein synthesis and degradation (Bard et al., 2019; Pohl and Dikic 2019).

Several PROTACs have been developed in the last 20 years, but unfortunately only few of them are selective for tumor cells. Many PROTACs recruit E3 ligases that are ubiquitously expressed in both normal and tumor tissues, thus leading to on-target toxicities. Multiple strategies can be followed to achieve the selective degradation of tumor-specific POIs mediated by PROTACs.

If the POI is tumor specific, it is possible to target it with any available E3s expressed in the tumor tissues (Burslem et al., 2019). Alternatively, if the POI is characteristic of a tumor-derived tissue, it is possible to optimize PROTACs taking advantages of any available tissue-specific E3 (Schapira et al., 2019; Sun et al., 2019). Further, a tumor-associated POI could be expressed in normal tissues and involved in physiological cell functions but showing an upregulated expression in cancer tissues. The use of tumor specific E3s highly expressed in tumor cells, but lowly or absent expression in normal tissues, could offer an increased advantage to selectively kill cancer cell, thus minimizing toxicity to normal tissues. The development of a B-cell lymphoma-extra-large (BCL-XL) PROTAC is a recent example (Chung et al., 2020; Kolb et al., 2021).

The availability of public -omics data has incentivized the identification of tissue-selective E3s (Consortium 2015; Melé et al., 2015) opening the route to achieve the selective and tumor specific degradation of a target protein by PROTACs.

Several research groups have recently investigated the activity of the light-controllable photo-PROTACs, which can be controlled under visible or UVA light to drive tumor specific degradation of POIs (Pfaff et al., 2019; Xue et al., 2019; Liu et al., 2020; Reynders et al., 2020). This strategy can only be accomplished in a clinical setting using photodynamic therapy for limited types of cancer.

PROTACs efficacy has been demonstrated in several preclinical studies (Bai et al., 2019; Khan et al., 2019; Li et al., 2019). Of note, PROTAC technology has also been shown to stimulate an anticancer immune response by inducing the presentation of peptides derived from the degradation of POI to antigen-presenting cells (Moser et al., 2017; Jensen et al., 2018). Moreover, PROTAC could be used to generate new MHC-I peptides on the cell surface favouring the formation of new immunopeptidome “targetable” by T-cell based therapeutics (Lai et al., 2018). Mass spectrometry analysis can help to understand and explore the impact of PROTAC treatment on peptide repertoire of MHC-I presentation and potential perturbation of biological pathways.

PROTAC strategy can be used to exploit E3s having tumor suppressor natural substrates (Hines et al., 2019), as well as PROTAC-incorporation into nanoparticles which can be incapsulated with antibodies, can help to specifically reach the tumoral environment and malignant cells (Beck et al., 2017; Niza et al., 2019; Pillow et al., 2020).

Recently, strategies similar to PROTACs have been developed to induce the degradation of RNAs (i.e., oncogenic micro-RNAs) through the recruitment of nucleases. These molecules, known as ribonuclease Targeting Chimeras (RIBOTACs) stands as innovative future anticancer therapeutics (Costales et al., 2020a; Costales et al., 2020b). Overall, PROTACs and similar technologies stand as promising class of biological drugs useful in cancer therapy.

### PROTACs as Therapeutic Option for Glioblastoma

Central nervous system (CNS) cancers are a group of heterogeneous tumor entities with wide differences regarding the site of onset, molecular biology, clinical behaviour, and etiology (Kristensen et al., 2019; Lospinoso Severini et al., 2020). Among them, glioblastoma (GB) is the most malignant and lethal in adults (Louis et al., 2016). Classified as grade IV diffuse glioma by the World Health Organization (WHO), GB encompasses more than 54% of gliomas with an median survival of about 15 months (Ostrom et al., 2014; Louis et al., 2016). Current standard therapy for newly diagnosed GB is based on maximal surgical resection, followed by radiation and chemotherapy, based on the administration of temozolomide (TMZ), an oral alkylating agent (Stupp et al., 2005; Stupp et al., 2009). Despite the aggressiveness of this therapeutic strategy, it has limited effectiveness making GB an incurable tumor that often returns as relapse (Lieberman 2017). The main hallmarks of this malignancy that hinder its treatments are rapid progression, invasiveness of cancer cells in the surrounding region of the brain, inter- and intra-tumoral genetic and molecular heterogeneity and the presence of drug-resistance GB stem-like cells (GSCs), which favour tumor relapse (Brennan et al., 2012; Meyer et al., 2015; Gangoso et al., 2021).

Transcriptomic and genomic profiling have allowed the identification of genetic alterations patterns affecting molecular drivers involved in GB tumorigenesis, including epidermal growth factor receptor (*EGFR),* phosphatase and tensin homolog (*PTEN*), cyclin dependent kinase 4/6 (*CDK4/6*) and cyclin dependent kinase inhibitor 2A/B (*CDKN2A/B*), neurofibromatosis type 1 (*NF1*), platelet-derived growth factor receptor alpha (*PDGFRα*)*,* and isocitrate dehydrogenase (*IDH*) genes (Verhaak et al., 2010; Dunn et al., 2012; Stoyanov and Dzhenkov 2018).

The delineation of the aberrant molecular networks that cause the malignant phenotype of GB have highlighted key processes, which can be therapeutically exploited. So far, several targeted therapies for GB have been tested, most of which aim to block growth factor receptors (i.e., EGFR) and downstream pathways frequently altered in GB (i.e., PI3K/AKT/mTOR and MAPK/ERK) (Le Rhun et al., 2019). However, none of these approaches have been formally validated as effective in clinical trials, likely due to molecular compensatory mechanism, insufficient target coverage or toxicity (Touat et al., 2017; Le Rhun et al., 2019). Different immunotherapeutic approaches have also been investigated for the treatment of GB, but the presence of the tumor immunosuppressive microenvironment limits their benefits (Bufalieri et al., 2020a; Weenink et al., 2020; Bufalieri et al., 2021; Medikonda et al., 2021).

Recently, the UPS is emerging as a promising source for the development of new therapeutic options for GB, and in particular PROTACs represent an interesting targeted therapy for the treatment of this devastating tumor (Bufalieri et al., 2020b; Scholz et al., 2020; Maksoud 2021; Farrell and Jarome 2021).

Two different PROTAC strategies able to induce the degradation of CDK4 and/or CDK6 have been tested in GB cells. CDK4 and CDK6 are crucial for cell cycle regulation and are attractive targets for the treatments of various types of cancers, including GB, frequently characterized by a CDK4/6 pathway dysregulation (Network 2008; Brennan et al., 2013; Bronner et al., 2019). In 2019, Zhao and Burgess tested the activity of PROTACs based on two selective CDK4/6 inhibitors, palbociclib (Ibrance^®^, Pfizer, New York, USA) and ribociclib (Kisqali^®^, Novartis, Basel, Switzerland) in breast cancer and GB cell lines (Zhao and Burgess 2019). These drugs have been approved by US Food Drug Administration (FDA) as combination therapy for ER-positive, HER2-negative advanced breast cancer and are currently used in ongoing clinical trials, including some for the treatment of GB (NCT03158389; NCT02345824; NCT02933736; NCT03834740; NCT03355794; NCT03355794). PROTACs of palbociclib and ribociclib (called pal-pom and rib-pom, respectively) consist in the conjugation of these two drugs to pomalidomide (pom), a cereblon (CRBN) E3 ligand, by cycloadding a known azide derived from pomalidomide to N-propargyl derivatives of palbociclib or ribociclib. U87 GB cells treated with pal-pom and rib-pom at 20–200 nM have a significant depletion of CDK4 protein levels, showing the effectiveness of these PROTACs to counteract the aberrant overexpression of this kinase in GB (Zhao and Burgess 2019). In addition, Su and others designed and synthesized a PROTAC by linking the CDK6 inhibitor palbociclib and E3 CRBN recruiter pom, testing its effect in GB cells (Su et al., 2019). In this study Nutlin-3b, VH032, and bestatin were also used as recruiting moiety for the E3 ligases Mdm2 and VHL, and inhibitor of apoptosis (cIAP), respectively. Interestingly, the authors found that in U251 GB cells CDK4 and CDK6 were degraded only with PROTAC recruiting CRBN, but not the other E3s, and that CDK4 degradation was less significant compared to those of CDK6. Furthermore, CDK6 degraders with shorter linker possessed higher degradation capacity, favouring the recruitment of CRBN towards CDK6 (Su et al., 2019). Although in-depth studies on the biological effect and anti-tumor potential of these PROTACs are still needed, these data suggest the potential application of PROTAC technology for the specific CDK4/6 degradation for the treatment of GB.

The first *in vivo* evidence of the potential of PROTACs as anticancer agents for GB was provided by a recent work in which the authors exploited the ability of a high-selective histone deacetylase 6 (HDAC6) inhibitor, J22352, to impair GB tumor growth (Liu et al., 2019). Indeed, the overexpression of HDAC6 in GB is associated with proliferation and resistance to TMZ, thus targeting this enzyme stands as a promising strategy for GB therapeutic interventions (Wang et al., 2016). J22352 shows PROTAC-like property, leading to the ubiquitylation and subsequent proteasome degradation of HDAC6. As consequence, the decrease of HDAC6 expression level significantly inhibits GB tumor growth in U87MG cells, both *in vitro* and *in vivo,* by increasing autophagic cancer cell death and eliciting the anti-tumor immune response (Liu et al., 2019).

These pioneering studies on the effects of PROTACs in GB cells and the evidence that PROTACs are already developed against oncoproteins relevant for the progression of this tumor, including EGFR (Zhang et al., 2020; Zhao et al., 2020), mitogen-activated MAP-kinases (MAPs) (Pandey et al., 2016; Trauner and Shemet 2019) and bromodomain and extraterminal (BET) protein BRD4 (Xu et al., 2018; Yang et al., 2019; Hu and Crews 2021; Yang et al., 2021), suggest the great potential for the use of this technology for the treatment of GB.

## Discussion

In the last two decades, targeting UPS has emerged as an extraordinary clinical opportunity, leading to the development of new and effective therapeutic options in human diseases, especially in cancer.

**FIGURE 2:**
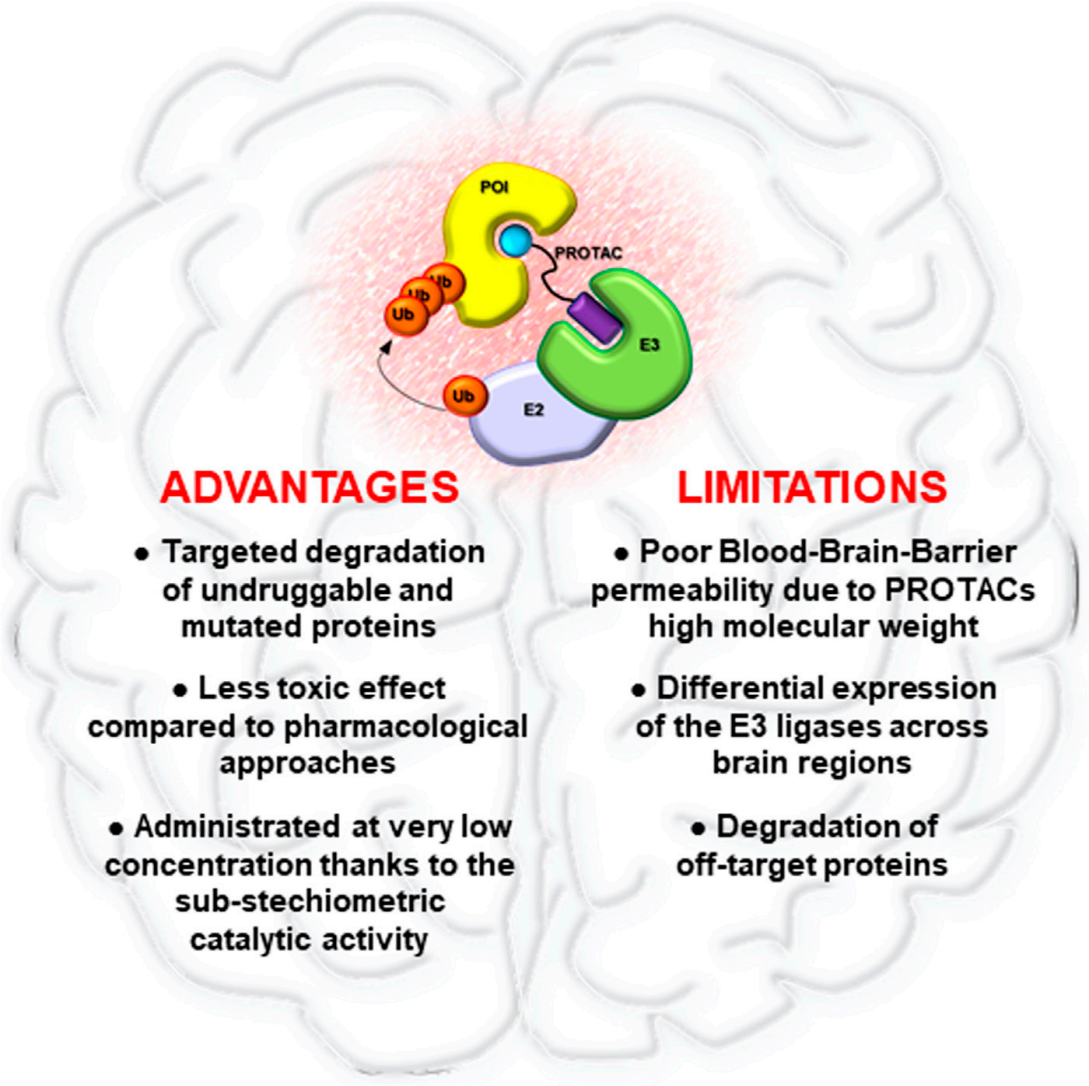
Advantages and limitations of PROTAC technology in brain tumors. Pro and cons of PROTACs application for the treatment of brain malignancies.

In this field, PROTAC has been one of the first strategies developed, aimed to degrade rather than inhibit protein targets. Thanks to their mechanism of action, PROTACs have shown the peculiarity to improve current cancer therapies based on the use of SMIs. Indeed, while SMIs act by occupying pockets on target proteins in a stoichiometric manner, a single PROTAC molecule can induce the degradation of its target through many rounds, even after dissociation of the PROTAC from POI (Lai and Crews 2017). This mechanism of action provides several advantages (Figure 2). Foremost PROTACs can be administered at lower dosages compared to SMIs achieving comparable effects, thus reducing toxicity. Moreover, PROTACs are less sensitive to drug resistance compared to traditional drugs. Indeed, PROTACs are potentially able to degrade multiple subunits of a protein complex, thus reducing the possibility to develop resistance-mutations in the protein of interest (Hu and Crews 2021). However, genomic alterations in the components of the E3s complex can cause resistance to PROTACs, underling the urgent need to find novel ligands for other druggable E3 ligases (Ottis et al., 2019; Zhang et al., 2019).

Given that many PROTACs targets are proteins involved in oncogenic proliferation and metastasis, PROTAC technology rapidly moved from laboratory to clinics especially for the treatment of human cancers (Zeng et al., 2021). At present, two Phase II clinical trials for the PROTACs ARV-471 and ARV-110 are ongoing, for the treatment of breast and prostate cancer, respectively. ARV-471 is an orally available PROTAC developed by Arvinas for the targeting of ER and its mutated forms, ER^Y537S^ ER^D538G^, resistant to endocrine therapy in ER-positive breast cancer (Martin et al., 2017). ARV-110, another orally available PROTAC, selectively degrades AR and inhibits pancreatic tumor growth, both in mice models and patient-derived organoids, better than enzalutamide, a known AR inhibitor (Neklesa et al., 2019). ARV-110 have been tested in Phase I clinical trial for castration-resistant prostate cancer (CRPC) and a Phase II clinical trial is ongoing to evaluate its pharmacokinetics and pharmacodynamics as well as its safety and tolerability, in CRPC patients (Petrylak et al., 2020).

Despite the rapid preclinical development of PROTACs as novel cancer therapeutics, many aspects need to be addressed. One of the biggest challenges is that PROTACs have high molecular weights, often larger than 1,000 Da, which could limit their cell permeability, pharmacokinetic abilities, oral bioavailability, and their capability to bypass the blood-brain barrier (Figure 2). In particular, this last aspect could represent a relevant limit for the clinical application of PROTACs in brain tumors, for which it will be essential to improve drug delivery systems for PROTACs, such as nano-vehicles, active transporter or alternative administration regimens (Banks 2016; Dong 2018).

One of the biggest weaknesess in the development of new PROTACs is the lack of knowledge for many E3s, especially regarding their tissue-specific expression and correlation to human diseases. So far, only a few E3s and ubiquitin ligase binders have been explored for the design of PROTACs. This aspect raises the need to study the biological functions and expression of E3 ligases as well as to solve their structures to accelerate the synthesis of new PROTACs. Moving forward, chemo-proteomic platforms, DNA-encoded library screening, and fragment-based ligand discovery will be useful both for the identification of E3s tissue, tumor, or compartment specific, and of ligands for incurable disease-related targets (Jacquemard and Kellenberger 2019). Despite the use of small molecule binders of only a few E3s, a fast progress has been made in this field, set the ground for a bright future of PROTACs in drug discovery and precision medicine. Overall, PROTAC technology shows unique advantages and great therapeutic potentials, thus possibly revolutionizing drug development and providing clinical benefits.
